# Crystal Structure of *Helicobacter pylori* Pseudaminic Acid Biosynthesis N-Acetyltransferase PseH: Implications for Substrate Specificity and Catalysis

**DOI:** 10.1371/journal.pone.0115634

**Published:** 2015-03-17

**Authors:** Abu I Ud-Din, Yu C. Liu, Anna Roujeinikova

**Affiliations:** 1 Department of Microbiology, Monash University, Clayton, Victoria, Australia; 2 Department of Biochemistry and Molecular Biology, Monash University, Clayton, Victoria, Australia; NCI-Frederick, UNITED STATES

## Abstract

*Helicobacter pylori* infection is the common cause of gastroduodenal diseases linked to a higher risk of the development of gastric cancer. Persistent infection requires functional flagella that are heavily glycosylated with 5,7-diacetamido-3,5,7,9-tetradeoxy-*L-glycero-L-manno*-nonulosonic acid (pseudaminic acid). Pseudaminic acid biosynthesis protein H (PseH) catalyzes the third step in its biosynthetic pathway, producing UDP-2,4-diacetamido-2,4,6-trideoxy-β-*L*-altropyranose. It belongs to the GCN5-related N-acetyltransferase (GNAT) superfamily. The crystal structure of the PseH complex with cofactor acetyl-CoA has been determined at 2.3 Å resolution. This is the first crystal structure of the GNAT superfamily member with specificity to UDP-4-amino-4,6-dideoxy-β-*L*-AltNAc. PseH is a homodimer in the crystal, each subunit of which has a central twisted β-sheet flanked by five α-helices and is structurally homologous to those of other GNAT superfamily enzymes. Interestingly, PseH is more similar to the GNAT enzymes that utilize amino acid sulfamoyl adenosine or protein as a substrate than a different GNAT-superfamily bacterial nucleotide-sugar N-acetyltransferase of the known structure, WecD. Analysis of the complex of PseH with acetyl-CoA revealed the location of the cofactor-binding site between the splayed strands β4 and β5. The structure of PseH, together with the conservation of the active-site general acid among GNAT superfamily transferases, are consistent with a common catalytic mechanism for this enzyme that involves direct acetyl transfer from AcCoA without an acetylated enzyme intermediate. Based on structural homology with microcin C7 acetyltransferase MccE and WecD, the Michaelis complex can be modeled. The model suggests that the nucleotide- and 4-amino-4,6-dideoxy-β-*L*-AltNAc-binding pockets form extensive interactions with the substrate and are thus the most significant determinants of substrate specificity. A hydrophobic pocket accommodating the 6’-methyl group of the altrose dictates preference to the methyl over the hydroxyl group and thus to contributes to substrate specificity of PseH.

## Introduction

*Helicobacter pylori* is a Gram-negative, microaerophilic bacterium that colonizes the stomachs of more than half of world’s population [1]. *H*. *pylori* infections are associated with a number of gastroduodenal disorders ranging from gastritis, gastric and duodenal ulcers to gastric adenocarcinoma and mucosa-associated lymphoid tissue lymphoma [2, 3]. It was the first bacterium to be classified as a group I (definite) carcinogen for human gastric cancer by the International Agency for Research on Cancer [4]. *H*. *pylori* has a unipolar bundle of two to six sheathed flagella that enable the bacteria to drill into the highly viscous mucus lining of the stomach and reach the gastric epithelium [5]. Flagella-mediated motility is required not only for initial colonization but also for attaining robust infection and persistence of *H*. *pylori* in the high-flow and rapid-turnover environment of the stomach [6, 7]. *H*. *pylori* flagellins are O-glycosylated on serines and threonines with an unusual nine-carbon sugar pseudaminic acid (Pse) that has only been found in bacteria. Flagellin glycosylation is essential for assembly of flagellar filaments and motility, and hence for virulence [8, 9]. Therefore, the Pse biosynthesis pathway can be a potential target for novel therapeutics.

**Fig 1:**
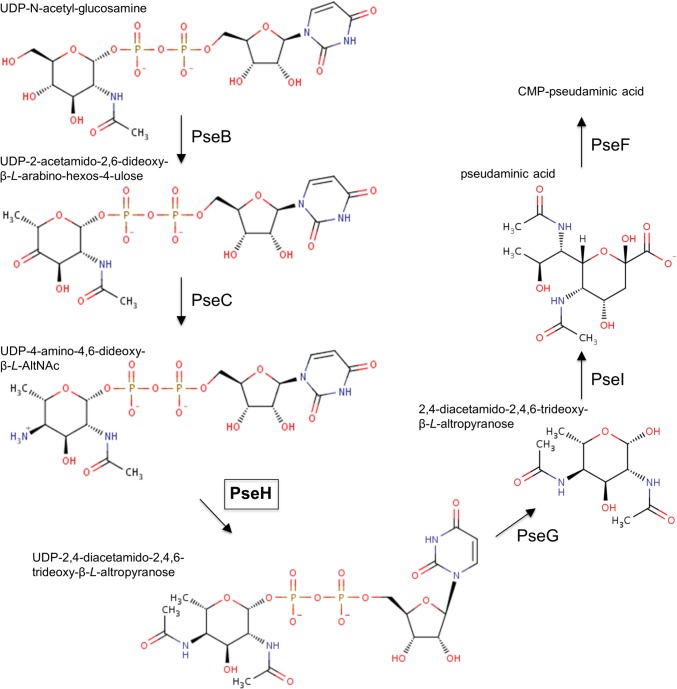
The CMP-pseudaminic acid biosynthesis pathway in *H*. *pylori* [10].

The first two enzymes in this pathway in *H*. *pylori*, UDP-N-acetylglucosamine dehydratase PseB and a pyridoxal-5-phosphate-dependent aminotransferase PseC, convert UDP-N-acetylglucosamine to UDP-4-amino-4,6-dideoxy-β-*L*-AltNAc (Fig. 1) [10]. The latter acts as a substrate for the 21-kDa Pse biosynthesis protein H (PseH), also known as flagellin modification protein H (FlmH) or flagellar protein G (FlaG1) [10, 11]. PseH is N-acetyltransferase (EC 2.3.1.202) that catalyzes transfer of an acetyl group from acetyl-CoA (AcCoA) to the C4 amino group of the nucleotide-linked sugar to produce UDP-2,4-diacetamido-2,4,6-trideoxy-β-*L*-Alt. Mutation in the *pseH* gene of the closely related species *Campylobacter jejuni* resulted in a non-motile phenotype that lacked flagella filaments and hook structures, indicating that PseH plays an essential role in flagella assembly [12].

Analysis of the PseH primary structure revealed low-level similarity to the GCN5-related N-acetyltransferase (GNAT) superfamily that covers more than 10,000 different enzymes from all kingdoms of life [13–15]. Members of the GNAT superfamily catalyze transfer of an acetyl group from AcCoA to the primary amine of a wide variety of substrates, including aminoglycosides, histones, arylalkylamines, glucosamine-6-phosphate, spermine, spermidine and serotonin [15]. Previous structural studies revealed that although different enzymes of this superfamily show only moderate pairwise sequence homology, they share a common core fold comprising a central highly curved mixed β-sheet flanked on both sides by α-helices, with the topology β0-β1-α1-α2-β2-β3-β4-α3-β5-α4-β6 [15–21].

The proposed reaction mechanism of most of the GNAT superfamily enzymes involves direct acetyl transfer from AcCoA without an acetylated enzyme intermediate [15]. In the first reaction step, a (non-conserved) general base abstracts a proton from the primary amine of the substrate to produce a lone pair of electrons, which then perform a nucleophilic attack on the thioester acetate. This leads to the formation of a transient bisubstrate intermediate that decomposes through proton transfer from a general acid (conserved tyrosine or serine) [15].

Limited structural information is available on enzymes that are functionally homologous to PseH. Acetyl transfer from AcCoA to the 4-amino moiety of the nucleotide-linked sugar substrate in a different biosynthetic pathway leading to legionaminic acid in *C*. *jejuni* is catalyzed by PglD which has a left-handed β-helix (LbH) fold and shows no detectable sequence similarity to PseH [22]. A different example of a bacterial nucleotide-sugar N-acetyltransferase, the *Escherichia coli* dTDP-fucosamine acetyltransferase WecD, belongs to the GNAT superfamily but shares only 15% sequence identity with PseH [17].

Here, we report the crystal structure of the *H*. *pylori* PseH complex with AcCoA solved at 2.3 Å resolution, which allowed us to address the molecular details of substrate binding and catalysis of this enzyme. This is the first crystal structure of the GNAT superfamily member with specificity to UDP-4-amino-4,6-dideoxy-β-*L*-AltNAc.

## Materials and Methods

### Purification, determination of the oligomeric state, crystallization, preparation of derivatives and data collection

Recombinant PseH from *H*. *pylori* was purified as previously described [14]. The oligomeric state of PseH in solution was determined by passing it through a Superdex 200 HiLoad 26/60 gel-filtration column (GE Healthcare) equilibrated with 50 mM Tris/HCl pH 8.0, 200 mM NaCl and calculating the molecular weight (MW) using a calibration plot of log MW versus the retention volume [V_retention_ (ml) = 549.3–73.9 × log MW] available at the EMBL Protein Expression and Purification Core Facility website http://www.embl.de/pepcore/pepcore_services/protein_purification/chromatography/hiload26-60_superdex200/index.html. The PseH-AcCoA crystal complex was obtained by co-crystallization with 5 mM AcCoA as described [14]. The crystals belong to space group I2_1_2_1_2_1_ with unit-cell dimensions *a* = 107.8 Å, *b* = 145.6 Å, *c* = 166.2 Å and three protein subunits in the asymmetric unit. Two different mercury derivatives were obtained by soaking crystals overnight in either mercury chloride (1 mM) or mercury potassium iodide (1 mM). To perform data collection at cryogenic temperatures, the crystals were briefly soaked in a cryo-stabilizing solution containing 1.0 M di-ammonium tartrate, 0.1 M sodium acetate trihydrate pH 3.8, 20% (v/v) glycerol and 5.0 mM AcCoA, and flash-frozen by plunging them into liquid-nitrogen. X-ray diffraction data for the native crystal were collected to 2.3 Å resolution using the MX2 beamline of the Australian Synchrotron. Diffraction data for the mercury chloride-derivitized crystal were collected to 2.4 Å resolution using the Australian Synchrotron MX1 beamline. Diffraction data for the mercury potassium iodide-derivitized crystal were collected to 2.8 Å resolution using the in-house Rigaku MicroMax-007 microfocus rotating-anode generator. All diffraction data were processed and scaled using *iMOSFLM* [23] and AIMLESS [24] from the CCP4 software suite [25]. Data collection statistics are summarized in Table 1.

**Table 1:** X-ray data collection and processing statistics.

**Data set**	**Native**	**HgCl_2_**	**K_2_HgI_4_**
**Wavelength (Å)**	0.95	1.01	1.01
**Resolution (Å)**	2.3 (2.4–2.3)	2.4 (2.5–2.4)	2.8 (3.0–2.8)
**Total No. of observed reflections**	168,694 (12,801)	385,414 (56,182)	117,999 (16,375)
**No. of unique reflections**	54,332 (4,535)	51,674 (7,446)	32,747 (4,705)
**Completeness (%)**	94 (96)	99 (99)	99 (99)
**Redundancy**	3.0 (2.8)	7.5 (7.5)	3.6 (3.5)
**Mean I/σ(I)**	10.3 (2.3)	15.9 (5.4)	11.9 (3.5)
**R**_**merge**_ ^**a**^	0.06 (0.30)	0.08 (0.35)	0.08 (0.31)
**Overall *B* factor from Wilson plot (Å^2^)**	34	27	25

Values in parentheses are for the highest resolution shell.

^a^ $R_{\mathrm{merge}}=\frac{\left(\sum_{h}\sum_{i}\left|I_{hi}-\langle I_{h}\rangle \right|\right)}{\sum_{h}\sum_{i}\left|I_{hi}\right|}$, where *I_hi_* is the intensity of the *i*th observation of reflection *h*.

### Structure determination

The structure of PseH was determined using the method of multiple isomorphous replacement coupled with anomalous scattering (MIRAS). The locations of the four Hg sites for the mercury chloride derivative and seven sites for the mercury potassium iodide derivative were found using Autosol [26] from the PHENIX software suit [27]. The overall figure of merit of the resulting phase set was 0.24 for data between 30 and 2.4 Å. An initial partial model generated using AutoBuild within PHENIX was manually completed using COOT [28] and then refined against the 2.3 Å resolution native data set using PHENIX. The electron density indicated that one acetate ion was bound to each PseH subunit. A complete model including water molecules, AcCoA and acetate ions was built through iterative cycles of re-building with COOT and refinement with PHENIX. Analysis of the stereochemical quality of the model was accomplished using MOLPROBITY [29]. The final refined model of the PseH-AcCoA complex contains 532 of the expected 555 amino acid residues, three acetate ions, three AcCoA molecules and 228 water molecules (R-factor 0.178, R_free_ 0.218). All the non-glycine residues lie in permitted regions of the Ramachandran plot with 97% of these in the most favoured regions. Refinement statistics are given in Table 2. Structure figures were prepared using PYMOL [30]. Accessible surface area was calculated using AREAIMOL from the CCP4 software suite [25] with a probe radius of 1.4 Å.

**Table 2:** Refinement statistics.

**Resolution range (Å)**	30–2.3
**Reflections**	54302
**Atoms**	4902
^**a**^ **R-factor**	0.178
^**b**^ **R**_**free**_	0.218
**Bond-length deviation from ideality (Å)**	0.008
**Bond-angle deviation from ideality (°)**	1.1
**Molprobity scores**	
**Ramachandran regions (%)**	
**Favored**	97
**Allowed**	3
**Outliers**	0
**Clashscore**	4.0
**Average B (protein atoms) (Å^2^)**	40
**Average B (water molecules) (Å^2^)**	43
**Average B (acetate ions) (Å^2^)**	42
**Average B (AcCoA) (Å^2^)**	67

^a^ $R=\frac{\sum_{h}\left|\left(\left|F_{\mathrm{obs}}\right|-\left|F_{\mathrm{calc}}\right|\right)\right|}{\sum_{h}\left|F_{\mathrm{obs}}\right|}$

^b^ The free R-factor was calculated on 5% of the data omitted at random.

### Protein Data Bank accession number

The coordinates of the PseH complex with AcCoA have been deposited in the Protein Data Bank (RCSB) under accession code 4RI1.

## Results and Discussion

### Overall structure of PseH and comparison to other members of the GNAT superfamily

Although unliganded PseH did not crystallize, co-crystallization with AcCoA readily yielded crystals. The structure of recombinant *H*. *pylori* PseH (residues 1–180 plus an additional GIDPFT fragment (cloning artifact)) was determined to 2.3 Å resolution by using the multiple isomorphous replacement coupled with anomalous scattering (MIRAS) method with two mercury derivatives. The asymmetric unit contains three molecules. To determine the correct oligomeric assembly, we performed size-exclusion chromatography and analysis of the packing of individual subunits in the crystal. When subjected to gel filtration, the protein eluted as a single peak with an apparent molecular weight of approximately 36 kDa, indicating that PseH behaves as a dimer in solution. In line with this, analysis of probable assemblies in the crystal using the PDBe PISA server (http://www.ebi.ac.uk/msd-srv/prot_int/cgi-bin/piserver) also suggested that PseH likely exists as a stable dimer in solution; two of the three molecules in the asymmetric unit form a non-crystallographic dimer, and the third molecule forms a similar dimer with a symmetry-related neighbor. The dimer is stabilized by an interface with a surface area per monomer (∼1,000 Å^2^) that is approximately 10% of the total surface area of a single monomer (∼10,100 Å^2^).

**Fig 2:**
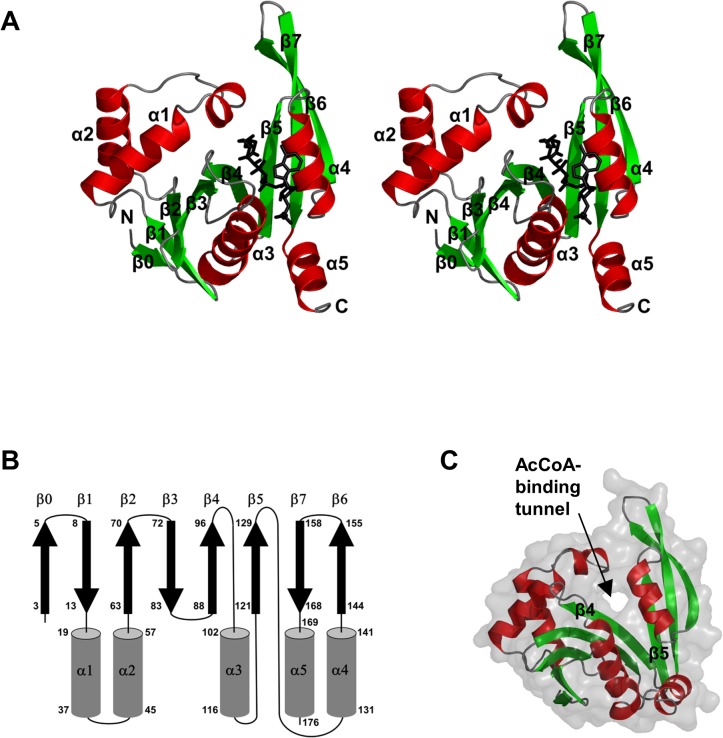
The overall fold of *H*. *pylori* PseH. (A) Stereo diagram of the structure of the PseH monomer. β-strands and α-helices are represented as arrows and coils and each element of the secondary structure is labeled and numbered as in text. The bound AcCoA molecule is shown in black. (B) The topology of secondary structure elements PseH. The α-helices are represented by rods and β-strands by arrows. Residue numbers are indicated at the start and end of each secondary structure element. (C) The molecular surface representation of PseH showing the AcCoA-binding tunnel between strands β4 and β5, which is a signature of the GNAT fold.

The PseH structure has a central twisted seven-stranded β-sheet flanked by five α-helices (Fig. 2A). The β-strands and α-helices are arranged in the topological order ββααβββαβαββαThe β-strands form a β-sheet in the order 01234576 (Fig. 2B). Strands β4 and β5 are splayed apart, creating a channel through the molecule (Fig. 2C) which is a signature of the GNAT fold. Helices α1 and α2 pack against one face of the β-sheet, helices α3 and α4 against the other, whereas helix α5 forms a C-terminal extension of strand β7.

**Fig 3:**
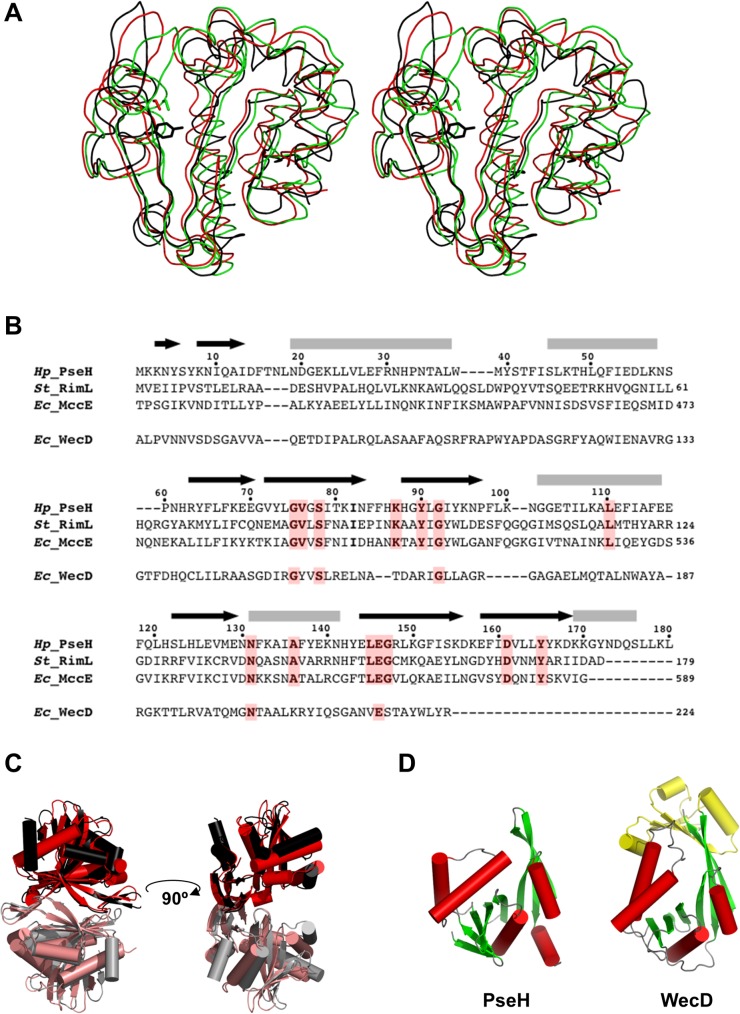
Comparisons of PseH with other GNAT superfamily enzymes. (A) Stereo ribbon diagram of the superimposed structures of PseH from *H*. *pylori* (black), RimL from *S*. *typhimurium* (red) and the acetyltransferase domain of MccE from *E*. *coli* (green). The side chains of the conserved tyrosine in PseH and serine in MccE and RimL, likely to be implicated in deprotonation of the leaving thiolate anion of CoA in the reaction, are shown using a stick representation. (B) A sequence alignment of PseH, RimL, MccE and WecD from *E*. *coli*. The elements of the secondary structure and the sequence numbering for PseH are shown above the alignment. Conserved residues are highlighted in red. (C) Comparison of dimers observed in the crystal structures of PseH (in which the two halves of the dimer are drawn in black and grey) and RimL (red/salmon). (D) Comparison of the structures of PseH and WecD. Like PseH, WecD catalyses transfer of an acetyl group from AcCoA to the 4-amino moiety of the nucleotide-linked sugar substrate. Structurally equivalent domains are drawn in the same colour. The additional N-terminal domain in WecD is shown in yellow.

In a comparison of PseH against the structures in the RCSB Protein Data Bank [31] that have been described in the literature, using the protein structure comparison service Fold at European Bioinformatics Institute (http://www.ebi.ac.uk/msd-srv/ssm) [32], significant similarities were found with other members of the GNAT superfamily. PseH has the closest structural similarity to *E*. *coli* microcin C7 self immunity acetyltransferase MccE (PDB ID code 3R9G [16]) and *Salmonella typhimurium* ribosomal protein L12 N^α^-acetyltransferase RimL (PDB ID code 1S7N [19]) (rms deviation of 2.4 Å and 2.2 Å for the superimposition of 160 C_α_ atoms (Fig. 3A), showing 18% and 14% sequence identity over equivalenced positions). MccE acylates the product of unwanted processing of the antibiotic microcin C7 in *E*. *coli*, thus inactivating it [16]. RimL possesses the same activity as MccE [33] and, in addition, converts the ribosomal protein L12 to L7 by acetylating its N-terminal amino group [19]. PseH, RimL and the acetyltransferase domain of MccE adopt a very similar fold, despite the limited sequence homology (less than 8% global sequence identity and less than 15% identity for pairwise alignments of PseH with MccE and RimM; see Fig. 3B). Structural similarity extends over the entire fold and includes all the secondary elements, except an additional C-terminal helix α5 in PseH (Fig. 3A). Furthermore, the mode of dimerization of PseH in the crystal is very similar to that of RimL [19] (Fig. 3C), although the second closest homologue (the acetyltransferase domain of MccE) is monomeric [16].

Further structural comparisons show that the PseH fold is very similar to the other members of the GNAT superfamily. Structural conservation of the GNAT fold has been related to its function as a scaffold for residues essential for AcCoA binding and catalysis [15]. In this respect, it is interesting to note that the structure of PseH is more similar to the GNAT enzymes that utilize amino acid sulfamoyl adenosine (MccE, RimL) or protein (RimL) as a substrate than a different GNAT-superfamily bacterial nucleotide-sugar N-acetyltransferase of the known structure, the *E*. *coli* dTDP-fucosamine acetyltransferase WecD (Fig. 3D) [17]. Like PseH, WecD transfers an acetyl group from AcCoA to the 4-amino moiety of the nucleotide-linked sugar substrate. Structural comparison shows that WecD contains an extra 70-amino-acid domain at the N-terminus (Fig. 3D) and a different number and order of strands in the β-sheet of the GNAT-domain, 2345617 (as compared to 01234576 in PseH). Alignment of the structures of PseH and the GNAT-domain in WecD resulted in a match of only 124 Cα atoms with rms deviation of 2.9 Å and 10% identity over equivalence positions.

A common mechanism of the acetyl transfer in GNAT enzymes involves protonation of the leaving thiolate anion of CoA by a general acid [15]. Previous mutagenesis studies were consistent with the role of Ser553 in MccE (structurally equivalent to Ser141 in RimL) as the general acid in catalysis [34]. In the superimposed structures of PseH, the MccE acetyltransferase domain and RimL, the side chain of Tyr138 of PseH is positioned close to that of Ser553 in MccE and Ser141 in RimL (Fig. 3A). Further structural superimpositions show that Tyr138 is structurally conserved in many GNAT superfamily transferases, including PA4794 from *Pseudomonas aeruginosa* (PDB ID 4KOW [18]), GNA1 from *Saccharomyces cerevisiae* (PDB ID 1I1D [20, 35]), sheep serotonin N-acetyltransferase (PDB ID 1L0C [36]) and human spermidine/spermine N1-acetyltransferase (PDB ID 2JEV [21]), where its role as a general acid in catalysis has been confirmed by mutagenesis. This suggests that Tyr138 acts as a general acid in the PseH-catalysed reaction.

### Binding of AcCoA and localization of the putative active site

**Fig 4:**
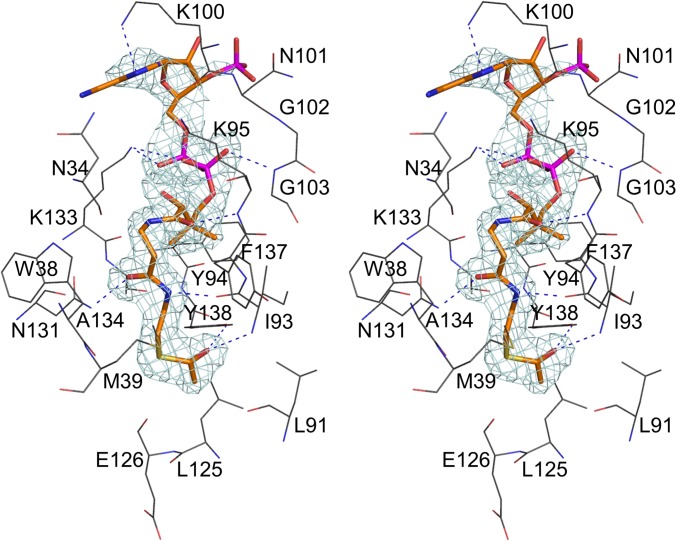
The stereoview of the electron density for AcCoA bound in the active site of PseH. The cofactor molecule is shown in CPK representation and coloured according to atom type, with carbon atoms in orange, nitrogen in blue, oxygen in red, phosphorus in magenta and sulphur in yellow. Only the protein residues that form hydrogen bonds or van der Waals contacts with the cofactor molecule are shown for clarity. Protein carbon atoms are coloured black. The hydrogen bonds important for recognition of the cofactor are shown. The map was calculated at 2.3 Å resolution with coefficients |F_obs_| − |F_calc_| and phases from the final refined model with the coordinates of AcCoA deleted prior to one round of refinement. The map is contoured at 3.0-σ level.

Analysis of the difference Fourier map revealed an AcCoA binding site between the splayed strands β4 and β5, which is the common cofactor site of GNAT superfamily enzymes (Figs. 2A and 4) [15–21]. The density for the entire molecule was readily interpretable, although somewhat less defined for the 3’-AMP moiety (Fig. 4). The position and extended conformation of AcCoA was found to be very similar to that described for other GNAT enzymes. The acetyl group of AcCoA is located at the bottom of the active site pocket on the face of the molecule opposite the AcCoA binding site. The pocket is lined with polar and aromatic residues. The carbonyl group of the thioester forms a bifurcated hydrogen bond with the main-chain amide of Ile93 (strand β4) and the hydroxyl of Tyr138, the putative general acid catalyst in the reaction. The acetyl moiety of AcCoA is further stabilized by van der Waals contacts with Leu91, Leu125 and Glu126. The β-alanine and β-mercaptoethylamine moieties are hydrogen bonded to the main-chain carbonyl of Ile93 (strand β4) and the side-chain of Asn131, and also interact through van der Waals contacts with Asn34, Trp38, Met39, Tyr94 and Ala134. The carbonyl oxygen of the pantoic acid moiety forms a hydrogen bond with the main-chain amide of Lys95 (strand β4), while the pyrophosphate group is stabilized by hydrogen bonds to the main chain of Gly103 and the side-chain of Lys133. The pattern of hydrogen bonds between the pantetheine moiety of AcCoA and strand β4 resembles bonding interactions in an antiparallel β-sheet, which is a common feature of GNAT enzymes.

### Model for UDP-4-amino-4,6-dideoxy-β-*L*-AltNAc binding and implications for catalysis

**Fig 5:**
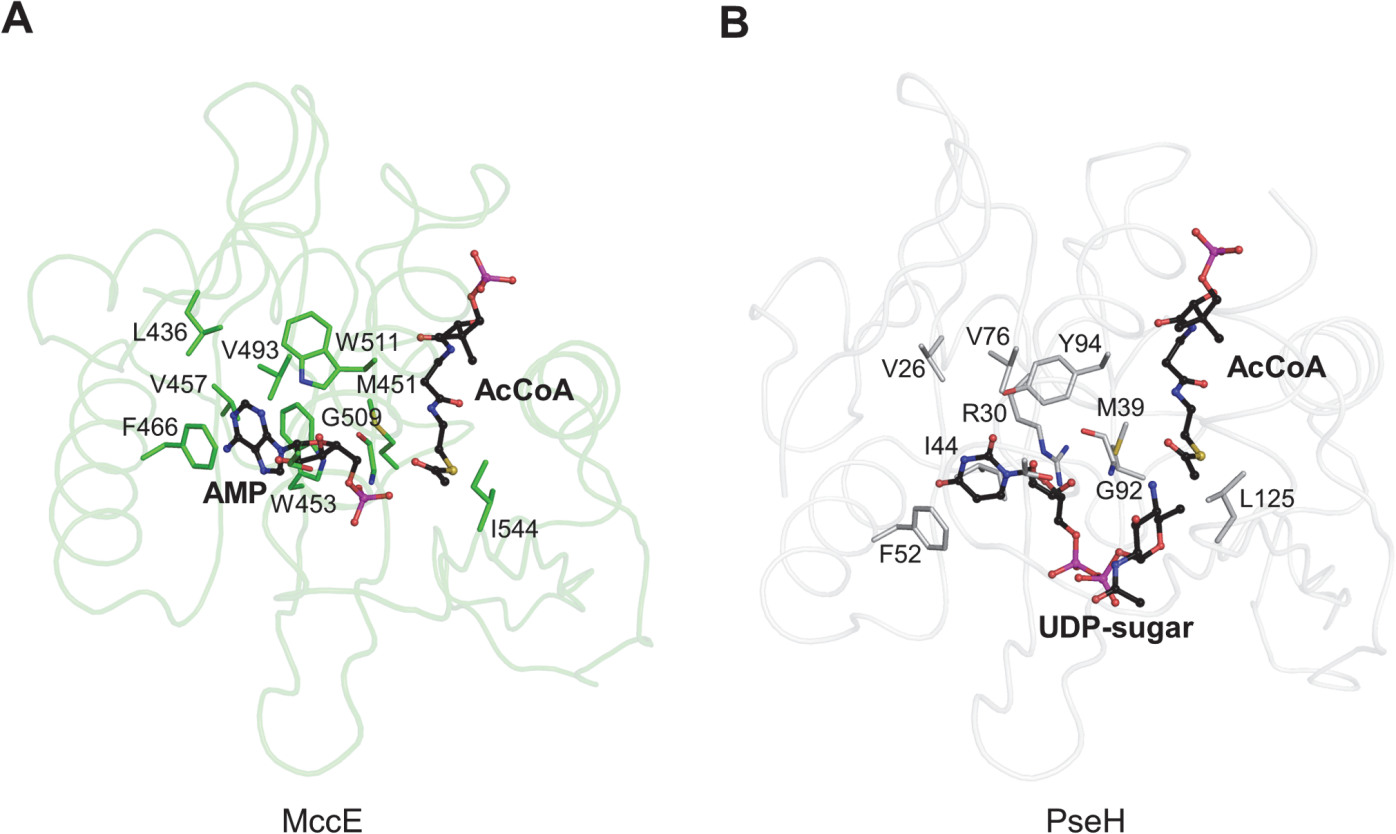
The structural similarity between the nucleotide-binding pocket in MccE and the putative nucleotide-binding site in PseH. The positions of the protein side-chains that form similar interactions with the nucleotide moiety of the substrate and with AcCoA are shown in a stick representation. The 3'-phosphate AMP moiety of CoA is omitted for clarity. (A) Key interactions between the protein and the nucleotide in the complex of the acetyltransferase domain of MccE with AcCoA and AMP. The protein backbone is shown as ribbon structure in light green for clarity of illustration. The AMP and AcCoA molecules are shown in ball-and-stick CPK representation and coloured according to atom type, with carbon atoms in black, nitrogen in blue, oxygen in red, phosphorus in magenta and sulphur in yellow. (B) The corresponding active-site residues in PseH and the docked model for the substrate UDP-4-amino-4,6-dideoxy-β-*L*-AltNAc. The protein backbone is shown as ribbon structure in light grey for clarity of illustration. AcCoA and modeled UDP-sugar are shown in ball-and-stick CPK representation and coloured according to atom type, with carbon atoms in black, nitrogen in blue, oxygen in red, phosphorus in magenta and sulphur in yellow.

The observed remarkable similarity between the overall folds of PseH, RimL and the acetyltransferase domain of MccE is consistent with their common ability to bind nucleotide-linked substrates. Indeed, analysis of the superimposition of the structures of PseH and the MccE acetyltransferase domain in complex with AcCoA and AMP revealed that the structural similarity extends to the architecture of the pocket that is occupied by the nucleotide moiety of the substrate in MccE (PDB ID 3R96 [16]) (Fig. 4A,B). In the crystal structure of the latter, the adenosine ring is sandwiched between Trp453 and Phe466, which are part of a largely hydrophobic pocket lined with residues change numbering here Leu436, Met451, Val493 and Trp511. Our analysis of the PseH structure revealed that many of the residues that form the corresponding pocket on the surface of PseH are structurally conserved between PseH and MccE. As Fig. 5 illustrates, the location and orientation of Val26, Met39, Phe52, Val76 and Tyr94 in PseH are similar to those of Leu436, Met451, Phe466, Val493 and Trp511 in MccE, respectively. The observed structural conservation of the nucleotide-binding pocket in PseH and MccE allowed us to model the nucleotide moiety of the UDP-4-amino-4,6-dideoxy-β-*L*-AltNAc substrate bound to PseH in a mode similar to that seen in MccE, with the uracil ring sandwiched between the side chains of Arg30 and Phe52 and forming face-to-face π-π stacking interaction with the aromatic ring of the latter (Fig. 5B).

**Fig 6:**
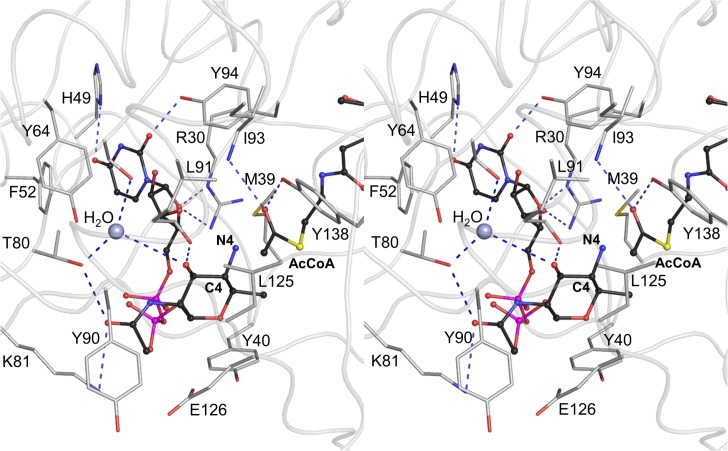
Interactions between the docked substrate UDP-4-amino-4,6-dideoxy-β-*L*-AltNAc, acetyl moiety of the cofactor and protein residues in the active site of PseH in the modeled Michaelis complex. The protein backbone is shown as ribbon structure in light grey for clarity of illustration. The substrate and AcCoA molecules are shown in ball-and-stick CPK representation and coloured according to atom type, with carbon atoms in black, nitrogen in blue, oxygen in red, phosphorus in magenta and sulphur in yellow. Only the protein side-chains that interact with the substrate are shown for clarity. The C4-N4 bond of the substrate (labeled) is positioned optimally for the direct nucleophilic attack on the thioester acetate, with the angle formed between the C4 of the amino-altrose, N4 of amino-altrose and the thioester carbonyl carbon being approximately 120°. The water molecule that is hydrogen bonded to the side-chains of Ser78 and Thr80, and is located within a hydrogen-bond distance of the 3’-hydroxyl of the modeled 4’-amino-altrose, is represented as a grey-blue ball. Deprotonation of the substrate’s amine group may occur via the 3’-hydroxyl of the altrose and this intervening water molecule.

Our structural analysis suggests that there are no residues in the vicinity of the AcCoA acetyl group that could serve as an acetyl acceptor and, thus, it is unlikely that the reaction proceeds through an enzyme-acetyl intermediate. The 4-amino-4,6-dideoxy-β-*L*-AltNAc moiety of the substrate has therefore been modeled next to the acetyl group of AcCoA, with the C4-N4 bond positioned optimally for the direct nucleophilic attack on the thioester acetate (Fig. 6) and in an orientation similar to that described for the functional homologue of PseH, WecD [17]. The model has been optimized to remove steric clashes and bring the bond length, bond angle and torsion angle values close to ideal by using the structure idealization protocol implemented in Refmac [37].

Analysis of this model (Fig. 6) suggests that the pyrophosphate moiety makes minimal contacts with the protein. In contrast, the nucleotide- and 4-amino-4,6-dideoxy-β-*L*-AltNAc-binding pockets form extensive interactions with the substrate and are thus the most significant determinants of substrate specificity. Calculations of the surface area of the uracil and 4-amino sugar rings shielded from the solvent upon this interaction give the values of 55% and 48%, confirming good surface complementarity between the protein and the substrate in the model. Hydrogen bonds between the protein and the substrate involve the side-chains of Arg30, His49, Thr80, Lys81, Tyr94 and the main-chain carbonyl of Leu91 (Fig. 6). Van der Waals contacts with the protein involve Met39, Tyr40, Phe52, Tyr90 and Glu126. Notably, the 6’-methyl group of the altrose points into a hydrophobic pocket formed by the side-chains of Met39, Tyr40, Met129 and the apolar portion of the β-mercaptoethylamine moiety of AcCoA, which dictates preference to the methyl over the hydroxyl group and thus to contributes to substrate specificity of PseH.

The proposed catalytic mechanism of PseH proceeds by nucleophilic attack of the 4-amino group of the altrose moiety of the substrate at the carbonyl carbon of the AcCoA thioester group. In our model of the Michaelis complex, the C4-N4 bond lies directly over the acetyl group with the angle formed between the C4 of the amino-altrose, N4 of amino-altrose and the thioester carbonyl carbon being approximately 120° (Fig. 6). The model is therefore consistent with the geometry of approach required for nucleophilic attack by the substrate. At physiological pH, the 4-amino group of the unbound substrate is positively charged [15]. How does PseH promote its deprotonation, converting it into a nucleophile? Our analysis of the crystal structure of the PseH/AcCoA complex and the model of the Michaelis complex shows that there are no titratable side-chains in the vicinity of the thioester group or the 4-amino group of the modeled substrate that could be directly involved in deprotonation. However, we note that all three PseH subunits in the asymmetric unit contain a well-ordered water molecule that is hydrogen bonded to the side-chains of Ser78 and Thr80, and is located within a hydrogen-bond distance of the 3’-hydroxyl of the modeled 4’-amino-altrose (Fig. 6). Deprotonation of the amine upon substrate binding may occur *via* this intervening water molecule, and identifies the conserved Ser78 as a putative general base in the reaction.

In summary, the first crystal structure of the GNAT superfamily member with specificity to UDP-4-amino-4,6-dideoxy-β-*L*-AltNAc presented here provides a molecular basis for understanding the third enzymatic step in the biosynthesis of pseudaminic acid in bacteria. The structure appears to be fully consistent with the mechanism that involves direct transfer of the acetyl group from AcCoA to the substrate. Our analysis pinpoints key structural features that might contribute to specificity of this enzyme and provides a useful foundation for more systematic mutagenesis and biochemical studies.
